# Health behaviours and fear of cancer recurrence in 10 969 colorectal cancer (CRC) patients

**DOI:** 10.1002/pon.4076

**Published:** 2016-02-11

**Authors:** A. Fisher, R.J. Beeken, M. Heinrich, K. Williams, J. Wardle

**Affiliations:** ^1^ Department of Epidemiology and Public HealthUniversity College LondonLondonUK

## Abstract

**Background:**

This study aimed to examine whether fear of cancer recurrence (FCR) was related to two important health behaviours (physical activity and smoking) in a large sample of colorectal cancer patients.

**Methods:**

Ten thousand nine hundred sixty nine patients, diagnosed in 2010–11, and in remission in 2013, completed the ‘Living with and Beyond Colorectal Cancer’ survey. The survey included purpose‐designed questions on fear of recurrence (‘*I have fear about my cancer coming back*’), demographics, treatment and health variables. Physical activity (PA) was recorded as number of days per week doing at least 30 min of brisk activity, and smoking status was reported.

**Results:**

Fifty per cent of respondents reported fear of their cancer returning. More women than men ((Odds Ratio; (OR) 1.58; 95% confidence interval (CI) 1.46, 1.71)) more younger than older patients (OR 2.53; CI 2.33, 2.74) and slightly more patients from deprived areas (OR 1.14, 1.05, 1.23) reported FCR. Independently of demographics and treatment, compared with those meeting the PA guidelines, those who were doing only ‘some’ (OR 1.22; CI 1.11, 1.35) or ‘no’ PA (OR 1.28; CI 1.15, 1.42) reported higher FCR. Compared with non‐smokers, more current smokers reported fear (OR 1.34, CI 1.10, 1.58) and slightly more ex‐smokers (OR 1.11; CI 1.04, 1.21).

**Conclusions:**

This cross‐sectional study provided novel data showing that colorectal cancer survivors with poorer health behaviours (those with lower activity levels and those who smoked) were more likely to experience FCR. Future research should replicate findings using detailed measures of fear, objective measures of health behaviours and identify directions of associations. © 2016 The Authors. Psycho‐Oncology Published by John Wiley & Sons Ltd.

## Introduction

Colorectal cancer is the fourth most common cancer in the UK, with around 40 000 new cases each year 1. Earlier diagnosis and improvement in treatments mean that 5‐year and 10‐year survival rates are now greater than 50% 2, so there is a focus on ensuring the best possible quality of life following diagnosis. Treatment of colorectal cancer usually involves surgical removal of the tumour, often with chemotherapy or radiotherapy. If the cancer returns following successful initial treatment, this is termed ‘cancer recurrence’ 3. The existing literature suggests that a significant proportion of cancer survivors suffer fear and anxiety that may substantially impair quality of life, even past 5‐year survival 4, 5. Indeed, fear of cancer recurrence (FCR) is commonly reported by survivors to be one of the most common unmet rehabilitation needs in their care pathway 6, 7.

There is emerging evidence that practising healthful behaviours (particularly being sufficiently physically active and not smoking) following a diagnosis of colorectal cancer may reduce the risk of recurrence. In a meta‐analysis of six prospective cohort studies examining post‐diagnosis physical activity, those who reported higher levels of physical activity following a diagnosis of colorectal cancer had significantly reduced risk of cancer‐specific mortality 8. A systematic review and meta‐analysis of 16 studies found that current smoking (and to a lesser extent former smoking) were associated with poorer prognosis 9. Given that FCR seems to be a key issue for people living with and beyond colorectal cancer, patients might be expected to make positive lifestyle changes. A number of theoretical models of health behaviour change, such as the Protection Motivation Theory and the Extended Parallel Processing Model, posit that perceptions of vulnerability to a health threat may motivate protective health behaviours 10, 11. However, there is little evidence that a cancer diagnosis alone acts a trigger for positive health behaviour change; a significant proportion of cancer survivors are insufficiently active, overweight and high‐risk drinkers, although a relatively low proportion report being smokers 12, 13. This suggests that patients need support in making lifestyle changes. Understanding the factors that relate to lifestyle and to FCR in colorectal cancer is an important step in designing comprehensive lifestyle interventions.

Most literature has focused on demographic factors relating to FCR (mainly in breast, head and neck, and prostate cancer survivors), generally demonstrating that fear is higher in women than men and higher in younger than older patients (reviewed in 14). Treatment types have been related to FRC, but findings are less consistent, probably reflecting the varying cancers studied 14. However, very few studies have examined associations between health behaviours and FCR in any group of cancer survivors.

In a repeat measures study of 73 patients with oral or oropharyngeal cancer, past and current tobacco use were associated with higher levels of psychological distress, and smokers reported higher FCR 15. In a longitudinal study of 154 thoracic or head and neck cancer patients, higher baseline FCR was associated with greater likelihood of continuing smoking 16. One study in 218 early stage breast cancer patients reported that higher fear was associated with higher frequency of GP visits, self‐examination and use of more counselling and support groups, but diet, physical activity, smoking or alcohol were not considered 17. A cross‐sectional study in 1336 long term survivors of testicular cancer also found smokers were more likely to experience FCR, but there was no association between fear and physical activity or alcohol consumption 18. In a cross‐sectional study of 7903 participants in the American Cancer Society's Study of Cancer Survivors II (mixed diagnoses, but the majority breast and prostate), FCR was positively correlated with a composite score of self‐rated positive health behaviour changes 19. To our knowledge, no quantitative studies have specifically examined the factors associated with FCR in survivors of colorectal cancer.

Therefore, the aims of this study were to use the largest existing relevant dataset of colorectal cancer patients to examine associations between FCR two important health behaviours. Associations between fear and socio‐demographic and treatment factors were also explored.

## Methods

### Procedure

Data were from the *Living with and Beyond Colorectal Cancer* survey, commissioned by the UK Department of Health in 2013 as part of a programme of work designed to ensure that UK cancer patients' needs are met across a range of health, psychosocial and lifestyle domains, through recording patient reported outcomes (PROMS) 20. The questionnaire was mailed by the National Cancer Registration Service (NCRS) to adult patients with a recorded diagnosis of colorectal cancer in 2010 or 2011 from hospitals around England and Wales (*n* = 34 467 were eligible). Permission to approach patients was granted to NCRS by the National Information Governance Board [ref ECC 5‐02(FT8)/12] and patients consented by returning completed questionnaires. Authors of the current study applied formally to NCRS for permission to perform secondary analyses on health and lifestyle data from the PROMS. Data were provided fully anonymised and authors had no access to any patient‐identifiable data. The current study was reviewed by NCRS and University College London, and in line with the University College London Ethics Committee, additional ethical approval was not required for secondary analyses of anonymous survey data. Content and face validity for the PROMS survey were identified through expert review and consultation with patients, experts and charity advisory groups 21.

## Measures

### Fear of cancer recurrence

The questionnaire section ‘Your Health and Wellbeing in the Past Month’ included the single‐item purpose‐designed question ‘*I have fear about my cancer coming back*’ (‘fear of recurrence’) with responses on a 5 point Likert scale (strongly disagree/disagree/neither agree or disagree/agree/strongly agree: coded 1–5) or ‘does not apply to me’ (coded as missing for the current study). The Living with and Beyond CRC survey was developed to audit multi‐dimensional aspects of care with a focus on quality of life and function. The current study involved secondary analyses of existing data, and a validated scale of FCR was not included in the survey because FCR and lifestyle were not a primary focus. However, the data gathered provide a unique opportunity to capture the views and behaviours of an extremely large and representative sample of CRC survivors. Additionally, much of the literature on FCR has used single‐item assessments of FCR, and findings are in accordance with more detailed validated scales (reviewed in 14).

### Health behaviours

Current physical activity was assessed by asking ‘*In the past week how many days have you done 30 minutes or more of brisk physical activity* (*This may include sport, exercise or brisk walking or cycling for recreation or to get to and from places, but should not include housework that or physical activity that is part of your job*)’. Responses were number of days from 0–7. Activity categories were ‘meeting guidelines’ (≥5 days per week), ‘some’ (1–4 days) and none (0 days). Smoking status was assessed using the question ‘*Do you consider yourself to be a smoker/ ex‐smoker / non‐smoker?*’.

### Demographics, treatment and health

Potential socio‐demographic and treatment confounders were selected a priori based on the existing literature on factors associated with FCR 14. Sex, age at diagnosis and ethnicity were recorded by patients. Socioeconomic status based on the Index of Multiple Deprivation score for home postcode was provided by the NCRS. Treatment questions included in the current study were ‘*How has your CRC responded to treatment*’ (‘fully responded I am in remission’, ‘has been treated but is still present’, ‘has not been treated’, ‘has come back after initial treatment’, ‘not certain what is happening’). Length of time since treatment was also assessed. Comorbidities were assessed with ‘*Do you have a long‐standing health condition other than cancer*’ (‘yes’, ‘no’, ‘can't say’). Virtually all patients reported having had surgery; however, in addition, they were asked to record whether they had received chemotherapy or radiotherapy.

## Analyses

For ease of interpretation, for main analyses, FCR data scores were categorised to those reporting no fear (categories 1–3) or reporting fear (categories 4–5). These analyses were repeated excluding category 3 (‘neither agree nor disagree’). However, because results did not change, these patients were included, giving approximately equal‐sized groups. Binary logistic regressions (fear/no fear as the outcome) were carried out, and simple associations were presented between demographics, health behaviours and treatment and health variables and fear presented. Adjusted models were also run for all demographics, both health behaviours adjusting for demographics, treatment and health and treatment and health adjusting for demographics. To illustrate the magnitude of the effect for health behaviours, graphs were also presented with FCR as a continuous outcome, using data analyses of covariance with polynomial contrasts.

## Results

The overall response rate to the survey was 63.3%, reported by Downing *et al*. 22. In the lifestyle dataset provided for the current study, 17 753 surveys were at least partially completed. Non‐responders were more likely to be from more deprived residential areas and more likely to be from the youngest and oldest age categories than non‐responders (*p* < 0.001). Ethnicity of non‐responders was not available for comparison, as this was gathered from the survey. Two hundred three participants who reported having Alzheimer's or dementia were excluded (because the questionnaire relied on participant recall) and only those patients who reported being in remission were included (*n* = 12,933). Of those, 602 (4.9%) recorded that FCR ‘did not apply to them’ (reasons for reporting this were not reported), leaving 11 686 patients. Ten thousand nine hundred sixty nine patients had complete data on all key outcomes for the current study.

Sample characteristics are presented in Table 1. 61% of the sample were men, 97% were white and 62% were ≥65 years old. Half of the patients (50%) reported some fear of their cancer returning. Twenty‐three per cent of patients met the guidelines for physical activity, 47% reported doing 1–4 days per week and 30% reported doing none. Five per cent were smokers, 40% ex‐smokers and 55% non‐smokers.

**Table 1:** Demographics, health behaviours and treatment associations with fear of cancer recurrence in 10 969 colorectal cancer patients

		%	Unadjusted OR (95% CI)	Adjusted OR (95% CI)
	*n*			
Demographics (A)				
*Sex*				
Male	3081	61.1	1	1
Female	4264	38.9	1.59 (1.48, 1.72)***	1.58 (1.46, 1.71)***
*Age*				
≥65	6772	61.7	1	1
<65	4197	38.3	2.53 (2.33, 2.74)***	2.52 (2.32, 2.73)***
*SES*				
Lower (less deprived)	5813	53.0	1	1
Higher (more deprived)	5156	47.0	1.14 (1.06, 1.23)***	1.14 (1.05, 1.23)***
Health behaviours (B)				
*Physical activity*				
Meeting guidelines	2495	22.7	1	1
Some	5159	47.0	1.26 (1.13, 1.39)***	1.22 (1.11, 1.35)***
None	3315	30.2	1.29 (1.17, 1.42)***	1.28 (1.15, 1.42)***
*Smoking*				
Non‐smoker	5978	54.5	1	
Ex‐smoker	4418	40.3	1.03 (0.95, 1.11)	1.11 (1.04, 1.21)**
Current smoker	573	5.2	1.44 (1.21, 1.72)***	1.34 (1.10, 1.58)**
Treatment (C)				
*Time since treatment*				
≥1 year	9574	12.1	1	1
<1 year	1314	87.9	1.47 (1.31, 1.66)***	1.26 (1.11, 1.43)***
Unknown/missing	81			
Radiotherapy				
No	8882	81.4	1	1
Yes	2027	18.6	1.25 (1.13, 1.37)***	0.95 (0.90, 1.12)
Unknown	60			
*Chemotherapy*				
No	5788	53.1	1	1
Yes	5121	46.9	1.83 (1.70, 1.97)***	1.54 (1.42, 1.68)***
Unknown	60			

SES (Socioeconomic status assessed by Index of Multiple Deprivation) adjusted model A included all demographics; adjusted model B included all health behaviours, demographics and treatment variables; adjusted model C included treatment variables and demographics. Models were run adjusting for presence of a comorbid condition and results did not change, so models without are presented (as comorbid condition had >1000 missing data points).

*** *p* ≤ 0.001.

** *p* = <0.005.

Factors associated with FCR are shown in Table 1. More women than men, more younger than older patients, and slightly more patients from deprived areas reported fear (*p* < 0.001). Associations with ethnicity could not be assessed because only 338 patients were from a non‐white ethnic group and of those, 182 classified themselves only as ‘other’. All significant associations between demographics and FCR remained in adjusted models. Treatment factors were related to FCR; patients who had more recently completed treatment were more likely to report fear (*p* < 0.001). In simple models, patients who received chemotherapy or radiotherapy were more likely to report fear (*p* < 0.001). However in adjusted models, only chemotherapy was significant.

**Figure 1:**
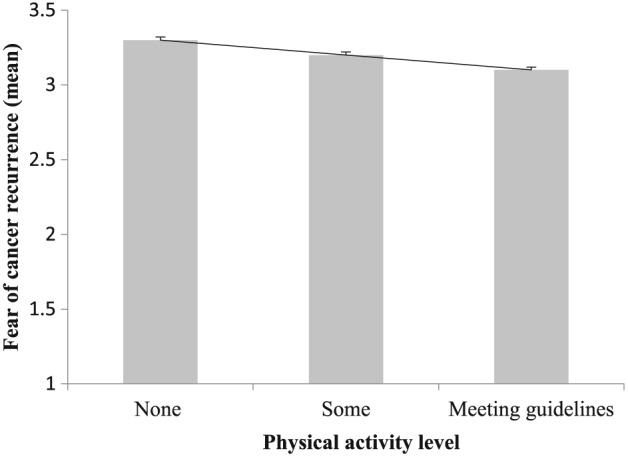
Fear of cancer recurrence by physical activity level in 10 969 colorectal cancer survivors. Values are means (adjusted for age, sex, socioeconomic status assessed by Index of Multiple Deprivation and treatment). Linear term is significant at *p* < 0.001. Activity levels are none (0 days/week of at least moderate activity), some (1–4 days) and meeting guidelines (5–7 days)

**Figure 2:**
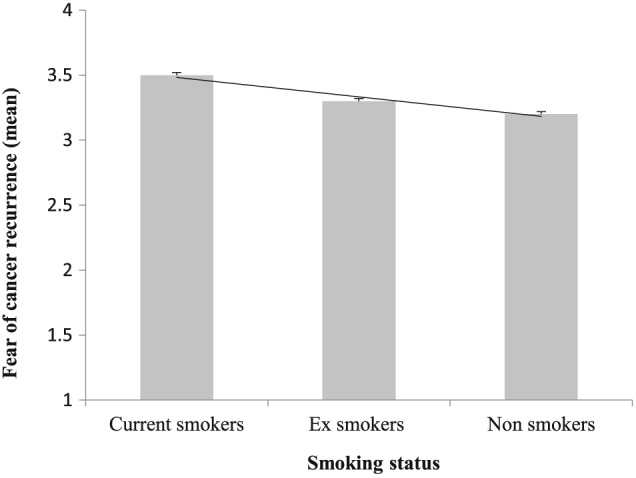
Fear of cancer recurrence by smoking status in 10 969 colorectal cancer survivors. Values are means (adjusted for age, sex, socioeconomic status assessed by Index of Multiple Deprivation, treatment and health). Linear terms is significant at *p* < 0.001

Associations between FCR and health behaviours are also shown in Table 1. After adjustment for multiple demographic and treatment confounders, when compared with those meeting the PA guidelines, patients who were doing some ((Odds Ratio (OR) 1.22, 95% Confidence Interval (CI) 1.11, 1.35, p < 0.001)) or no physical activity (OR 1.28, CI 1.15, 1.42 p < 0.001) reported higher levels of fear. When compared with never smokers, current smokers reported higher levels of fear (OR 1.34, CI 1.10, 1.58, p < 0.005), and ex‐smokers reported slightly higher levels (OR 1.11, CI 1.04, 1.21, p < 0.005). Figure 1 illustrates the continuous associations between physical activity level and FCR. There was a significant linear trend for higher levels of fear with lower levels of activity. Similarly, as shown in Figure 2, there was a significant association between smoking and FCR, such that fear was highest in current, then ex‐smokers then non‐smokers.

## Discussion

This was the first study to explore whether health behaviours related to FCR in colorectal cancer patients. We found that those with lower levels of PA, and current smokers, were more likely to experience FCR although the magnitude of those associations was relatively small. Additionally, this study adds to the literature demonstrating that demographic and treatment factors are related to fear.

In our study, current smokers (compared with never or ex‐smokers) were more likely to report fear. Although no studies in colorectal cancer survivors have previously examined associations between FCR and smoking status, comparable literature comes from head and neck cancer patients, where it has been observed that smokers report significantly higher levels of fear 15. It is plausible that the links between smoking and cancer are so widely publicised, and iterated by health professionals during the care pathway for patients undergoing curative treatment, that those who smoke have higher FCR. However, in a longitudinal study of 154 thoracic or head and neck cancer survivors, higher levels of fear undermined quit attempts 16, so FCR potentially should be addressed within stop smoking interventions in cancer survivors.

We found that patients who reported doing more physical activity reported slightly lower levels of FCR. The reasons for these findings are unclear. However, there is good evidence that physical activity reduces levels of general anxiety and depression and improves well‐being in cancer survivors 23, 24. There is also evidence that activity reduces some of the common symptoms of cancer and its treatments, such as fatigue 23, 24. Therefore, it is plausible that those who are more active are more generally able to cope and have a better psychological profile overall. It cannot be ruled out that patients with more disease‐related comorbidities or who had had more aggressive treatments were less likely to be active (and more likely to experience FCR), but in the current study, associations existed after adjustment for multiple confounders.

In line with theories of health behaviour change, it is also feasible that those who experience higher levels of fear or perceive themselves to be at higher risk of recurrence are more motivated to change their behaviour 10, 11. In the aforementioned study, 7903 US cancer survivors of mixed diagnoses were asked to rate whether they believed they were doing ‘more’, ‘less’ or ‘the same’ for 13 positive health behaviours, including physical activity. Higher FCR was associated with more positive health behaviour changes. Although overall, proportions reporting positive changes in behaviour were limited 19. Although promising, these analyses (and those in the current study) were cross‐sectional, using self‐reported measures. Therefore, further research examining associations between physical activity and FCR using objective measures and with a longitudinal design would be warranted.

To our knowledge, only one other study has reported the associations between physical activity levels and FCR. In a study of 1336 long‐term testicular cancer survivors, there was no association between reported physical activity level and FCR 18. The findings from our study suggest that any associations may be relatively weak, and plausibly in an all‐male sample with such long‐term follow‐up (>11 years), there was not enough variation in FCR to see association; indeed only a quarter of survivors reported fear. In line with our findings however, current smokers in this study reported significantly higher fear 18.

Fifty per cent of patients in our sample expressed some FCR, which adds to the large body of literature showing that FCR is common and pervasive in cancer survivors 14. Similarly, our finding that time since diagnosis was related has been demonstrated previously 14. There are very few studies specifically in colorectal cancer, but in qualitative sample of 81 male and female colorectal cancer patients, 82% reported at least some fear and believed that practising healthful behaviours may reduce risk of recurrence, although actual health behaviours were not reported 25.

The finding that more women than men, and younger than older participants, reported fear is consistent with a number of other studies, and the associations between fear and socioeconomic status that were observed in our study have been demonstrated in prostate, testicular and breast cancer survivors 14. However, ours was the first large study to examine these demographics in a large and representative sample of colorectal cancer patients, and the consistency with findings from other studies adds to this body of literature, as well as providing some support for the purpose‐designed single item measure using in the current study. Having had chemotherapy was related to higher FCR in colorectal cancer patients and this is consistent with other cancer types 14. This is perhaps unsurprising, because chemotherapy is usually associated with more advanced disease. The side effects are also potentially more impactful, so fear of having to receive such treatment in future could be higher. Qualitative studies with patients to untangle the reasons for these factors are required.

## Strengths and limitations

A clear and unique strength of the current study is the large population‐based sample of CRC survivors. However, there are limitations that must be acknowledged. The majority of the sample was from a white ethnic group, therefore results may not be generalisable to other ethnicities. The Living with and Beyond Colorectal Cancer PROMS were developed to gather data on a large number of aspects of life after diagnosis. The questionnaire was around 30 pages long, and therefore, it was not feasible to use validated scales to assess every item. As previously mentioned, in future research, where FCR is a key outcome, validated scales should be used, and there are now validated brief scales that could be used when questionnaire length is a consideration 26. Equally, a self‐reported one‐item assessment of physical activity was used, rather than objective measures or a validated scale. However, it can only be expected that associations may be stronger using validated items and given the lack of data on FCR in large populations, and virtually none examining associations with lifestyle, it seemed appropriate to capitalise on the valuable PROMS data as a ‘first step’. The magnitude of the associations between health behaviours in the current study were relatively small, reflecting around a 5% difference in those reporting FCR in those meeting PA guidelines versus not and in smokers versus non. Whilst associations may have been stronger with more detailed and validated measures, until this can be tested, the clinical importance of these associations will remain in question. However, there is enough evidence to recommend that increasing physical activity levels and stopping smoking are positive goals for cancer survivors and that FCR could be addressed in lifestyle interventions because survivors report this as a consistently unmet need 6, 7. We only had data on smoking and activity levels, but of course dietary patterns and alcohol are important health behaviours in colorectal cancer patients. Again, the length of the PROMS made detailed assessment of diet and alcohol unfeasible; however, future PROMS might consider including a brief assessments to gather at least a ‘snapshot’ of diet and alcohol. Five percent of the sample selected the ‘Does not apply to me’ option in response to the question about fear of their cancer recurring, rather than selecting an option on the Likert Scale. However, there was no additional data on why this option was selected. It is possible that respondents did not fully understand that their cancer, once treated, could return, or they were fear avoiders. In addition, it is possible that some participants may have selected this option if they did not experience fear (rather than choosing ‘strongly disagree’). Therefore, for this study, data from these participants was treated as missing. Further research could examine some of the factors that determine beliefs about cancer recurrence. Data from this study were cross‐sectional, so direction of associations between colorectal cancer and health behaviours could not be determined and may indeed be bidirectional.

## Conclusions

This study provided novel data showing that colorectal cancer survivors with poorer health behaviours (those with lower activity levels and those who smoked) were more likely to experience FCR. Further research should replicate findings using more detailed measures of fear, objective measures of health behaviours and aim to identify the direction of the associations.

## Conflict of interest

The authors have declared no conflicts of interest.
